# ORI-Explorer: a unified cell-specific tool for origin of replication sites prediction by feature fusion

**DOI:** 10.1093/bioinformatics/btad664

**Published:** 2023-10-31

**Authors:** Zeeshan Abbas, Mobeen Ur Rehman, Hilal Tayara, Kil To Chong

**Affiliations:** Department of Electronics and Information Engineering, Jeonbuk National University, Jeonju 54896, South Korea; Khalifa University Center for Autonomous Robotic Systems (KUCARS), Khalifa University, Abu Dhabi, United Arab Emirates; School of International Engineering and Science, Jeonbuk National University, Jeonju 54896, South Korea; Department of Electronics and Information Engineering, Jeonbuk National University, Jeonju 54896, South Korea; Advances Electronics and Information Research Center, Jeonbuk National University, Jeonju 54896, South Korea

## Abstract

**Motivation:**

The origins of replication sites (ORIs) are precise regions inside the DNA sequence where the replication process begins. These locations are critical for preserving the genome’s integrity during cell division and guaranteeing the faithful transfer of genetic data from generation to generation. The advent of experimental techniques has aided in the discovery of ORIs in many species. Experimentation, on the other hand, is often more time-consuming and pricey than computational approaches, and it necessitates specific equipment and knowledge. Recently, ORI sites have been predicted using computational techniques like motif-based searches and artificial intelligence algorithms based on sequence characteristics and chromatin states.

**Results:**

In this article, we developed ORI-Explorer, a unique artificial intelligence-based technique that combines multiple feature engineering techniques to train CatBoost Classifier for recognizing ORIs from four distinct eukaryotic species. ORI-Explorer was created by utilizing a unique combination of three traditional feature-encoding techniques and a feature set obtained from a deep-learning neural network model. The ORI-Explorer has significantly outperformed current predictors on the testing dataset. Furthermore, by employing the sophisticated SHapley Additive exPlanation method, we give crucial insights that aid in comprehending model success, highlighting the most relevant features vital for forecasting cell-specific ORIs. ORI-Explorer is also intended to aid community-wide attempts in discovering potential ORIs and developing innovative verifiable biological hypotheses.

**Availability and implementation:**

The used datasets along with the source code are made available through https://github.com/Z-Abbas/ORI-Explorer and https://zenodo.org/record/8358679.

## 1 Introduction

DNA replication in eukaryotes necessitates the precise synthesis of huge quantities of DNA, which is a vital element in ensuring genetic data integrity prior to cellular division (Baldauf 2003, Du *et al.* 2019, Abbas *et al.* 2022). As a cell divides, two DNA samples are dispersed to the daughter cells, and the genetic information is transferred to the progeny through cellular proliferation (Norris 2019). The position where DNA begins to replicate is known as the origin of replication sites (ORIs) (Cobb 2017). Genetically inherited mutation in the species may occur from abnormal replication. The precise DNA replication process assures the continuation of genetic data and the species’ reliability.

The DNA replication initiating mechanism consists mostly of highly controlled two stages: (i) origin licensing and (ii) origin activation (Barry and Bell 2006, Douglas *et al.* 2018). The DNA sequence and histone characteristics influence replication origin licensing and activation (MacAlpine and Almouzni 2013). Researchers have observed that 30 000–50 000 DNA replication origins are triggered during each cell cycle in mammals (Moiseeva and Bakkenist 2018). Yet, it is still unknown how replicating factors pick and recognize them. In prokaryotes, the replication beginning location is generally one, but in eukaryotes, numerous such locations are observed making it a complicated task (Bleichert *et al.* 2017, Dao *et al.* 2021, 2022). The recognition of ORIs is a critical step in the DNA replication process. As a result, identifying ORIs is essential to comprehend the control process of the DNA synthesis initiating phase and other biological development mechanisms. Moreover, it promotes medication research and cancer therapy.

Given the relevance of ORIs, various attempts have been made to forecast them. This study has concentrated on the efficient identification of ORIs through artificial intelligence (AI). AI-based approaches are more effective and less expensive than conventional experimental techniques for uncovering possible novel ORIs and leveraging hidden aspects of ORIs, which can aid researchers in comprehending the fundamental mechanism. Nowadays, various AI-based ORIs prediction algorithms have been created. Gao *et al.* created ori-finder, an online application for identifying ORIs in prokaryote genomes (Gao and Zhang 2008). Thereafter, they developed ori-finder2 (Luo *et al.* 2014), which uses an integrative approach to investigate additional details from several angles, such as the Z-curve technique and the FIMO tool that is used to locate the motif sequences. Dao *et al.* (2021) developed iORI-Euk, which combines several sequence-based features to train the Support Vector Machine to identify ORI sites.

Zhang *et al.* (2016) designed a predictor called iOri-Human, that uses physicochemical features along with the pseudo nucleotide composition features to detect ORI regions in the human species. Liu *et al.* (2018) created a predictor named iRO-3wPseKNC that can identify the characteristic of whole replicating zones of yeast species in order to make greater advantage of the CG asymmetric features. Furthermore, Singh *et al.* (2018) developed a classifier using content and contextual-based computational analysis. Lastly, Wei *et al.* (2021) proposed Stack-ORI framework, which uses 12 different feature descriptors to train the eXtreme Gradient Boosting (XGBoost) classifier (Chen *et al.* 2015) to predict ORI regions in four different species. From the literature, Stack-ORI is the most recent and efficient technique, which was created utilizing huge benchmark datasets for multiple species. This strategy performed well and effectively promoted the finding of prospective ORIs, though it requires considerable refinement as it did not consider neural network features at all.

We presented ORI-Explorer, a unique, intelligible, technique that uses a fused feature vector to train the CatBoost classifier for identifying cell-specific ORIs from several eukaryotic species. The ORI-Explorer uses four different feature descriptors, where one is extracted using the distinctive neural network architecture while the other three are composition *k*-spaced nucleic acid pairs (CKSNAP), Parallel Correlation Pseudo Dinucleotide Composition (PCPseDNC), and Dinucleotide-based Cross Covariance (DCC). While these features are concatenated and given to SHapley Additive exPlanation (SHAP) (Lundberg and Lee 2017) to select the most important features that are further used by CatBoost to predict the ORI regions. Cross-validation evaluations and independent testing revealed that the strategy of combining sequence features with neural network features outperforms the baseline model using individual feature descriptors and current predictors in terms of overall performance.

## 2 Dataset

To develop a trustworthy and accurate prediction model, it is essential to build an impartial and meticulous benchmark dataset. For this purpose, Dao *et al.* (2021) built a high-quality dataset and analysed their model, iORI-Euk. The researchers took the empirically validated ORI sequences with more than 300 bp from the Deori database (Gao *et al.* 2012). To eliminate the noise that was created by sequences of varying lengths, they extracted samples with a defined fixed length of 300 bp from the positive sequences. The equivalent negative samples were then isolated from 300 to 600 bp downstream and −600 to −300 bp upstream regions.

Since the datasets provided by iORI-Euk (Dao *et al.* 2021) are the most updated ones and to have a fair comparison with the previous SOTA models, we used the exact same datasets with same sequence lengths for model construction in the current work. Supplementary Table S1 provides a statistical summary of several cell-specific eukaryotic datasets.

## 3 Proposed architecture

**Figure 1.**
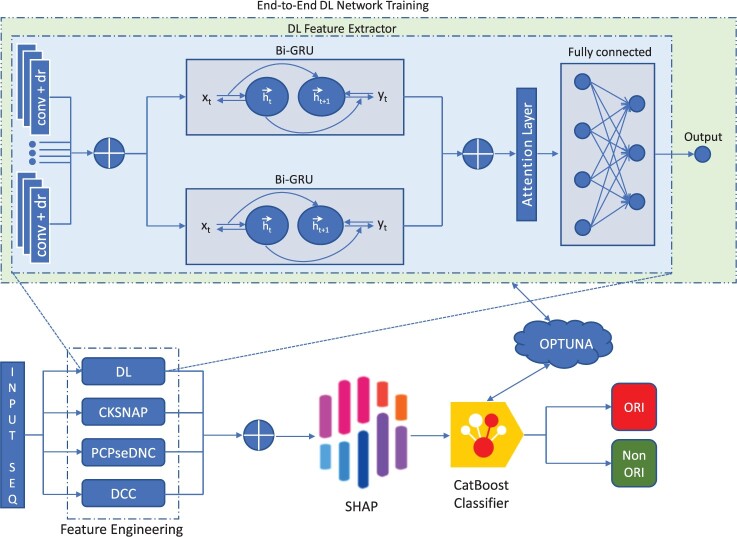
Network architecture of the proposed model, ORI-Explorer.

The proposed architecture is demonstrated in Fig. 1. The proposed architecture includes a feature engineering module (extracts features from input sequence), a feature optimization module (selects important features from the concatenated features), and a CatBoost Classifier module. These modules are discussed below.

### 3.1 Feature engineering module

Four different feature descriptors are computed and then concatenated to identify the ORIs. These feature descriptors are discussed in further subsections.

#### 3.1.1 Feature descriptor from deep learning module

The proposed neural network probes the fusion of bidirectional gated recurrent unit (GRU) (Chung *et al.* 2014), attention module, and convolutional layers with vacillating kernels and window sizes. The network comprises six convolutional layers, where input is fed to all the layers in a parallel configuration. Each convolution layer generates feature maps that contain the insight features of the input sequence. The convolution layers extract hierarchical features from the input data. Each successive layer is responsible to extract more abstract features from data, features having different-sized patterns are targeted to be extracted. Increasing the filters of the convolution layer helps to extract more abstract features from the data, further the receptive field can be enhanced by increasing the dimensions of the kernels. The non-linear activation function ReLU (Agarap 2018) is applied on every convolution layer. Each layer is followed by a dropout layer to prevent overfitting. As each layer has varied kernels and dimensions, it implies multiple hierarchical features are obtained and concatenated to form a strong feature representation.

The concatenated features are given to two different bidirectional GRUs containing different numbers of layers. The intention to use different numbers of layers is to make sure that the loss of data is prevented. Bidirectional GRU computes features and addresses the issue of vanishing gradients and generates lesser parameters in comparison to conventional long-short-term-memory (LSTM) (Hochreiter and Schmidhuber 1997). The feature vectors computed by both bidirectional GRUs are concatenated and given to the attention module.

As opposed to processing all inputs equally, ORI-Explorer uses an attention mechanism that enables the model to concentrate its attention on specific input components. A query, a set of key–value pairs, and a process for calculating a weighted sum of the values determined by the similarity among the query and the keys usually constitute the attention algorithm. A set of key–value pairs make up the attention layer’s input. The query, which in our case is a feature vector, is received by the attention layer. The attention layer uses dot product, to calculate a similarity measure among each key and the query. The softmax function is applied to the similarity scores to produce a set of weights that represent the relative importance of each value. The final achieved vector is given to a fully connected layer to get the prediction for the ORI sequence.

#### 3.1.2 CKSNAP

The CKSNAP encoding system calculates the frequency of occurrence of nucleotide pairs split by any *k* nucleotides. *k* is set to zero, one, two, three, four, or five. Using *k *=* *0 as an instance, we get 16 0-spaced dinucleotide pairings (AA, AC, AG, AT, CA, CC, CG, CT, GA, GC, GG, GT, TA, TC, TG, and TT). A feature vector for this can be expressed as,

(1) $$\mathrm{CKSNAP}=(\frac{C_{AA}}{C_{\mathrm{total}}},\frac{C_{AC}}{C_{\mathrm{total}}},\frac{C_{AG}}{C_{\mathrm{total}}},\ldots ,\frac{C_{TT}}{C_{\mathrm{total}}}).$$

Each descriptor’s value represents the content of the matching nucleic acid pair in the given sequence. For example, if in the sequence AC occurs *n* times, its content is equivalent to *n* divided by the entire number of 0-spaced nucleic acid pairs in the given sequence, which is $C_{\mathrm{total}}$.

#### 3.1.3 PCPseDNC

To understand PCPseDNC, first we need to understand PseDNC as the same concept applies to the PCPseDNC. Mathematically, PseDNC can be described as:

(2) $$C=[c_{1},c_{2},c_{3},\ldots ,c_{16},\ldots ,c_{16+\lambda }],$$

where

(3) $$c_{m}=\{\begin{array}{cc} \frac{f_{m}}{\sum_{i=1}^{16}f_{i}+w\sum_{j=1}^{\lambda }\theta_{j}} & \text{if};\;1\leq m\leq 16\\ \frac{w\theta_{m-16}}{\sum_{i=1}^{16}f_{i}+w\sum_{j=1}^{\lambda }\theta_{j}} & \text{if};\;17\leq m\leq 16+\lambda \end{array}.$$

Here, $f_{m}$ for 1 ≤m ≤ 16 is the standardized frequency of dinucleotides in the DNA sequence, λ denotes the correlation in the sequence with the best scored value, *w* is a weighting parameter ranged between 0 and 1, and $\theta_{j}$ (j=1,2,…,λ) is the *j*-tier correlation value defined as:

(4) $$\{\begin{array}{c} \theta_{1}=\frac{1}{L-2}\sum_{i=1}^{L-2}\theta (D_{i}D_{i+1},D_{i+1}D_{i+2})\\ \theta_{2}=\frac{1}{L-3}\sum_{i=1}^{L-3}\theta (D_{i}D_{i+1},D_{i+2}D_{i+3})\\ \theta_{3}=\frac{1}{L-4}\sum_{i=1}^{L-4}\theta (D_{i}D_{i+1},D_{i+3}D_{i+4})(\lambda <L)\\ \ldots \\ \theta_{\lambda }=\frac{1}{L-1-\lambda }\sum_{i=1}^{L-1-\lambda }\theta (D_{i}D_{i+1},D_{i+\lambda }D_{i+\lambda +1}) \end{array},$$

where the correlation function is defined as:

(5) $$\theta (D_{i}D_{i+1},D_{j+1}D_{j+2})=\frac{1}{\mu }\sum_{u=1}^{\mu }[P_{u}(D_{i}D_{i+1})-P_{u}(D_{j}D_{j+1}){]}^{2},$$

where μ indicates physiochemical indices, $P_{u}(D_{i}D_{i+1})$ is the value at uth index of dinucleotide $D_{i}D_{i+1}$ at index *i*, and $P_{u}(D_{j}D_{j+1})$ denotes the value of dinucleotide at index *j*.

The main difference between PseDNC and PCPseDNC is the number of physiochemical indices μ, where the former uses only 6 physiochemical indices while the latter uses 38 different physiochemical indices.

#### 3.1.4 DCC

The DCC encoding calculates the relationship between two independent physicochemical indexes at two dinucleotides that are isolated from one another along the sequence by lag nucleic acids. Mathematically, the DCC encoding is determined as follows:

$$\begin{array}{c} DCC(u_{1},u_{2},\mathrm{lag})=\sum_{i=1}^{L-\mathrm{lag}-1}(P_{u_{1}}(D_{i}D_{i+1})-{\bar{P}}_{u_{1}})\\ (P_{u_{2}}(D_{i+\mathrm{lag}}D_{i+\mathrm{lag}+1}-{\bar{P}}_{u_{2}})/(L-\mathrm{lag}-1). \end{array}$$

Here, $u_{1}$ and $u_{2}$ are unique physicochemical indexes, *L* indicates the length of the sequence, $P_{u_{a}}(D_{i}D_{i+1})$ is $u_{a}$’s numerical value for $D_{i}D_{i+1}$ dinucleotide at location *i*, and ${\bar{P}}_{u_{a}}$ is the estimated value at location $u_{a}$ throughout the sequence, which can be calculated mathematically as:

(6) $${\bar{P}}_{u_{a}}=\sum_{j=1}^{L-1}P_{u_{a}}(D_{j}D_{j+1})/(L-1).$$

The feature vector extracted using DCC has a size of N×(N−1)×LAG, where *N* is the number of distinct nucleotides and the max of lag is termed as LAG (lag=1,2,3,…,x).

### 3.2 Feature optimization module (SHAP)

SHapley Additive exPlanation is usually termed as SHAP, as it follows the intuition behind the game theory and uses the SHapley values to build ground explanations related to machine learning (ML) models. Its widespread utility is to find out the features, which are most consequential during the prediction of a sample by ML or deep learning (DL) models. Features and predictions are denoted as players and goals, while the payout is referred to as feature attributes. These SHAP values determine the role of each feature in a corresponding prediction of a data sample. A player can be a single feature or multiple features grouped together, which is quite usual when multiple regions are formed into super-region and the respective prediction is associated with them. The influence of a particular feature is determined by using SHAP values. SHAP explains the connection between the local values, which are model-based and SHapley values using an expression which is given below:

(7) $$g(q^{‘})=K_{0}+\sum_{t=1}^{n}K_{t}q_{t}^{‘}.$$

Where *n* is the max size of the fusion vector, *K* is the SHapley value as a feature attribute, the fusion vector is termed as *q*, and *t* are the features of the data sample. Features are grouped together to form a fusion vector. The value of one represents that a particular feature is present and zero means it is absent from the feature vector. In order to compute SHAP values certain features are set to be in existing status (playing) and some of them are set to down status (absent). The property of missingness states that a feature is assigned a zero value if it is missing. Prominent features are assigned large SHapley values. This signifies that SHAP is based on the measure of feature attributes. Kernel SHAP figures out the significance of features in a data sample and their importance to the final prediction. Tree Shap is utilized as an alternative to Kernel Shap for tree-based ML models, such as decision trees (DTs) and random forests. Kernel Shap follows these steps to find out the feature’s importance. Firstly, a feature fusion vector is formed where one is assigned to features that are present and zero otherwise.

(8) $$q_{k}^{‘}\in {\left(0,1\right)}^{n}.$$

**Figure 2.**
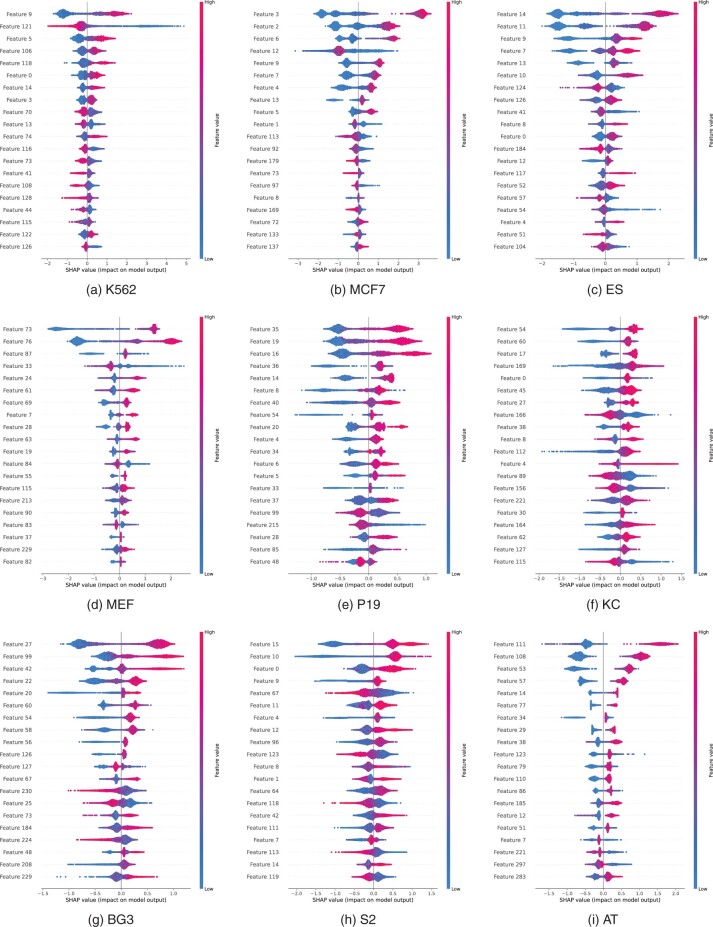
Top 20 important features calculated using SHAP for every cell. (a) K562, (b) MCF7, (c) ES, (d) MEF, (e) P19, (f) KC, (g) BG3, (h) S2, and (i) AT.

Further, the corresponding predictions are evaluated for each fusion vector, and then by utilizing the Kernel SHAP, the weights of all the fusion vectors are computed. The Sharpley values are finally obtained by fitting the weighted model. Figure 2 shows the feature importance of the top 20 features calculated using SHAP.

We combined the four encodings in the order: DL features, CKSNAP features, PCPseDNC features, and then DCC features. We stated the feature range in Supplementary Table S7, displaying which feature relates to which encoding.

### 3.3 CatBoost classifier module

In 2017, the Russian search engine company Yandex created the CatBoost ML model, a specific kind of gradient-boosting algorithm (Prokhorenkova *et al.* 2018). It is an improved variant of the Gradient Boosting Decision Trees (Ke *et al.* 2017) that makes use of an advanced ensemble learning method based on the gradient descent concept. A group of DTs are successively built during the feature learning process to produce each succeeding tree with a smaller loss. In other words, each DT produces a powerful learner by using the knowledge from the previous tree to affect the following tree and improve prediction accuracy.

The sequential boosting strategy of the CatBoost algorithm is an additional significant feature. In conventional GBTs, after performing a number of boosting processes, all the training data are given to build a predictor. Through this method, the built model experiences a prediction shift, which ultimately results in a unique form of target permeability (Hussain *et al.* 2021). By using the ordered boosting architecture, the CatBoost method solves the aforementioned issues. Furthermore, CatBoost offers a greater capacity to prevent the overfitting problem by providing an accurate estimation of the gradient step when compared to various regular boosting algorithms. It has also demonstrated better results (Prokhorenkova *et al.* 2018, Al Daoud 2019, Jaganathan *et al.* 2022) and speed on a variety of well-known ML tasks compared to the current state-of-the-art techniques, including XGBoost (Chen and Guestrin 2016) and LightGBM (Ke *et al.* 2017). Consequently, it appears to be the main justification for using it in the present research.

## 4 Model optimization

Developing an efficient and precise model requires the optimization of a ML framework. The optimization process improves the framework, which results in considerable performance gains. The algorithm may acquire more information from data and produce accurate decisions if the proper architecture is chosen. Therefore, in order to optimize different parameters of the proposed architecture, we have used the OPTUNA framework (Akiba *et al.* 2019).

### 4.1 OPTUNA

OPTUNA is an optimizing tool that performs hyper-parameter tuning for improving the ML framework. It is a Bayesian optimization-based framework that repeatedly creates a probability model of the functional mapping from hyper-parameter search space. The working principle of OPTUNA is as follows,

- Create a search space: the very first step is to establish the hyper-parameter search space that we wish to improve.
- Defining loss function: the next phase is to create a loss function that utilizes a hyper-parameter as input and produces a scalar number that needs to be optimized.
- Evaluation of loss function: multi-armed bandit technique is used to evaluate the loss function by effectively exploring the hyper-parameter space.
- Updating the probabilistic model: Optuna models the link among hyper-parameters and the loss function using Bayesian inference. It modifies the Bayesian framework according to loss function evaluations and computes the likelihood of improved performance for every set of hyper-parameters.
- Choosing the next set of hyper-parameters: Optuna chooses the next set of hyper-parameters to assess depending on the likelihood of gain. This procedure is continued until certain criteria, which might include a specific number of assessments or a given performance threshold, are fulfilled.
- Delivering the optimal hyper-parameters: after the optimization technique is over, Optuna provides the best collection of hyper-parameters discovered during the selection phase.

The parameter ranges used to optimize the model are tabulated in Supplementary Table S2, while Supplementary Tables S3 and S4 show the selected best parameters by OPTUNA.

## 5 Results

Utilizing the same datasets used by iORI-Euk (Dao *et al.* 2021) and Stack-ORI (Wei *et al.* 2021), we assessed the proposed models’ capability to distinguish ORIs, using both 10-fold cross-validation and independent testing datasets. Table 1 illustrates the outcomes of ORI-Explorer for 10-fold cross-validation while Table 2 displays the results for independent testing.

**Table 1.** 10-fold cross-validation performance achieved using ORI-Explorer.

Species	Cell	MCC	Ac	Sn	Sp	AUC	*F*1	Precision
*H.sapiens*	K562	0.773	0.886	0.859	0.912	0.9473	0.882	0.907
	MCF7	0.687	0.843	0.820	0.866	0.925	0.839	0.860
*M.musculus*	ES	0.793	0.896	0.891	0.902	0.955	0.895	0.899
	MEF	0.723	0.861	0.872	0.849	0.937	0.862	0.853
	P19	0.747	0.873	0.895	0.851	0.942	0.876	0.857
*D.melanogaster*	KC	0.781	0.890	0.909	0.871	0.952	0.892	0.876
	BG3	0.692	0.846	0.854	0.839	0.924	0.847	0.840
	S2	0.663	0.831	0.854	0.808	0.906	0.835	0.817
*A.thaliana*		0.90	0.950	0.954	0.946	0.986	0.949	0.946

**Table 2.** Independent testing performance achieved using ORI-Explorer.

Species	Cell	MCC	Ac	Sn	Sp	AUC	*F*1	Precision
*H.sapiens*	K562	0.770	0.884	0.851	0.917	0.950	0.880	0.911
	MCF7	0.675	0.836	0.791	0.881	0.919	0.828	0.869
*M.musculus*	ES	0.794	0.897	0.893	0.901	0.962	0.897	0.90
	MEF	0.721	0.861	0.875	0.846	0.942	0.862	0.850
	P19	0.752	0.876	0.891	0.861	0.941	0.878	0.865
*D.melanogaster*	KC	0.771	0.885	0.906	0.865	0.951	0.888	0.870
	BG3	0.699	0.849	0.854	0.846	0.929	0.850	0.847
	S2	0.664	0.832	0.86	0.803	0.907	0.836	0.814
*A.thaliana*		0.871	0.935	0.952	0.918	0.98	0.936	0.920

### 5.1 Comparison with existing methods on independent dataset

**Figure 3.**
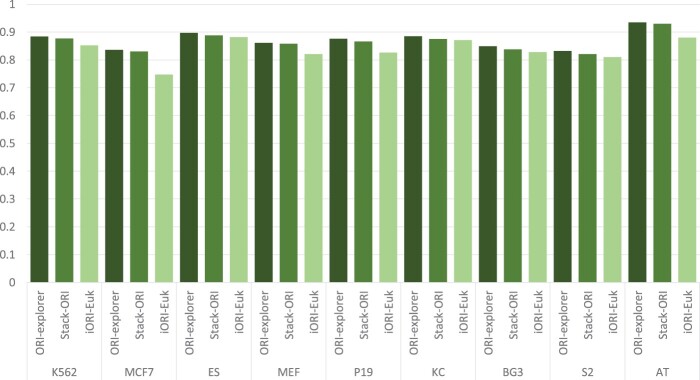
Performance comparison of ORI-Explorer with previous SOTA models based on accuracy.

**Figure 4.**
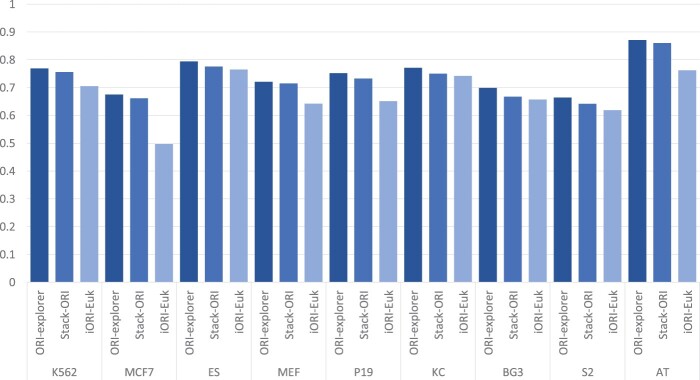
Performance comparison of ORI-Explorer with previous SOTA models based on MCC.

In general, a model may typically be overoptimized while training to increase its performance. An unseen dataset must be used to assess the constructed model in order to guarantee its stability or generalizability. We contrasted ORI-Explorer’s performance with that of the earlier SOTA models, iORI-Euk and Stack-ORI. Figures 3 and 4 demonstrate that ORI-Explorer is consistently superior to the previous models in terms of both accuracy and MCC. Compared to the previous SOTA models, ORI-Explorer achieved higher MCCs in the range of 1.1%–3.2%. The MCC is a single statistic that takes true positives, false positives, true negatives, and false negatives into account. Higher MCC indicates a better balance of sensitivity and specificity, implying that the model is not biased toward a single class and can make accurate predictions for both positive and negative samples. It also signifies a convincing predictive power of the model capturing profound patterns in the data. Therefore, the achieved results indicate that the ORI-Explorer is a stronger and reliable model that can be used for cell-specific ORI identification in a variety of species.

### 5.2 Cross-specie testing

**Figure 5.**
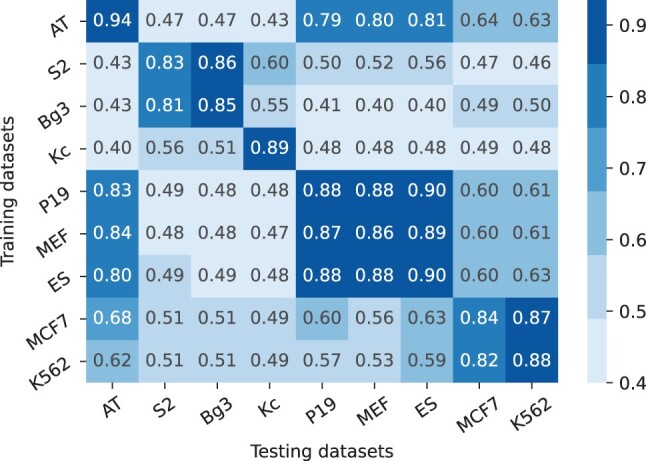
Cross-species performance using independent datasets based on accuracies.

We assessed the applicability of a specie- or cell-specific model to other cells. By placing the training dataset on the *y*-axis and the testing dataset on the *x*-axis, we validated the performance of all the cells on other cells and created the heatmap as shown in Fig. 5. The network performs better when tested against the same cell except for MCF7, MEF, P19, and S2. In the case of MCF7, it achieved an accuracy of 84% on itself and 87% on K562, in the case of MEF it performed a little better on ES. Similarly, P19 performed slightly better on ES, and S2 achieved slightly higher accuracy on Bg3. The important point to be noted here is, all these cells which performed slightly better on other cells belong to the same species. No cell from a specific specie performed better than a cell from another specie. So, from these achieved results, it is noticeable that distinct ORI-sequences preserve species specificity. Therefore, the aforementioned findings emphasize the significance of cell- and species-specific prediction models.

## 6 Ablation study

Numerous modules including the feature engineering module, feature optimization module, and the classifier were used to enhance the performance of the proposed model, ORI-Explorer. Ablation research is conducted in order to determine the significance of each subsystem.

### 6.1 Performance assessment on individual feature encodings

To show the importance of combining the four different feature descriptors, we compared the results achieved by each descriptor and tabulated the results in Supplementary Table S5. Seven out of nine cells including MCF7, ES, MEF, P19, BG3, S2, and AT achieved better performance on the DL module while K562 and KC achieved better performance on CKSNAP. By combining all four encoding the proposed architecture performed always better on all nine cells as presented in Table 2.

Since the DL module was performing the best, we made an assessment by removing the subsystems of the DL module as well.

### 6.2 Performance assessment without attention module

The attention method (Vaswani *et al.* 2017) is designed to retrieve global information and address the issue of long-term dependencies. It is an improvement of encoder–decoder architecture in natural language processing and several other applications. Due to its importance, researchers are using it for omics data as well (He *et al.* 2021, Li *et al.* 2021, Yao *et al.* 2023). By removing the attention module, we computed the performance for the *Homo sapiens* cells and noticed that the performance declined for K562 from 76.96% to 74.83% in terms of MCC and from 88.4% to 87.4% in terms of accuracy. Similarly, for MCF7 the MCC decreased by 6.27% and the accuracy by 3%. A complete comparison using all the evaluation metrics is presented in Supplementary Table S6.

### 6.3 Performance assessment without GRU modules

GRU is a subtype of Recurrent Neural Network, which is faster and needs less memory as compared to LSTM. Therefore, we used it in our DL feature descriptor module. As part of the ablation study, we removed the module and tested the models’ performance as stated in Supplementary Table S6. After removing the Bi-GRU modules, the MCC of the human cell K562 declined from 79.96% to 67.61% while the accuracy declined from 88.4% to 83.8%. Furthermore, for the cell MCF7, the MCC decreased by 10.55% and the accuracy was decreased by 5.15%. A complete comparison using all the evaluation metrics is presented in Supplementary Table S6.

## 7 Conclusion

Determining the appropriate ORIs is critical for a variety of biological fields, such as genetic engineering, cell genetics, and biotech. We created ORI-Explorer, a novel AI-based approach that combines three traditional feature-encoding techniques and a feature set obtained from a deep-learning neural network model. An optimum feature set is selected from the combined feature vector to train the CatBoost algorithm that predicts the ORIs. Cross-validation and independent testing validated that ORI-Explorer performs better than the existing state-of-the-art techniques. During the construction of ORI-Explorer, it is observed that choosing the CatBoost classifier lowers model biasness while increasing variability, resulting in improved generalization ability. Moreover, it is observed that deep neural networks do acquire important features from the DNA sequence for the identification of ORIs. But those features are not enough for efficient classification, therefore, the combination of features provides a better platform for ORIs detection. Additionally, SHAP, a model interpretive technique, was used to identify the most essential ORI classification elements. A web server is made openly available at https://nsclbio.jbnu.ac.kr/tools/ORI-Explorer/ to helps the larger scientific society. ORI-Explorer may be used to estimate ORI using the genomic sequence information in a high-throughput manner.

## Supplementary Material

btad664_Supplementary_Data

## References

[btad664-B1] Abbas Z , Tayara H, Chong KT. ZayyuNet- A unified deep learning model for the identification of epigenetic modifications using raw genomic sequences. *IEEE/ACM Trans Comput Biol Bioinform* 2022;19:2533–44. 10.1109/TCBB.2021.3083789. 34038365

[btad664-B2] Agarap AF. Deep learning using rectified linear units (ReLU). arXiv, arXiv:1803.08375, 2018, preprint: not peer reviewed.

[btad664-B3] Akiba T , Sano S, Yanase T et al Optuna: a next-generation hyperparameter optimization framework. In: *Proceedings of the 25th ACM SIGKDD International Conference on Knowledge Discovery & Data Mining, Anchorage, AK, USA, August 4–8, 2019*. 2019, 2623–31. ACM.

[btad664-B4] Al Daoud E. Comparison between XGBoost, lightGBM and CatBoost using a home credit dataset. Int J Comput Inf Eng 2019;13:6–10.

[btad664-B5] Baldauf SL. The deep roots of eukaryotes. Science 2003;300:1703–6. 12805537 10.1126/science.1085544

[btad664-B6] Barry ER , Bell SD. DNA replication in the archaea. Microbiol Mol Biol Rev 2006;70:876–87. 17158702 10.1128/MMBR.00029-06 PMC1698513

[btad664-B7] Bleichert F , Botchan MR, Berger JM. Mechanisms for initiating cellular DNA replication. Science 2017;355:eaah6317. 28209641 10.1126/science.aah6317

[btad664-B8] Chen T , Guestrin C. Xgboost: a scalable tree boosting system. In: *Proceedings of the 22nd ACM SIGKDD International Conference on Knowledge Discovery and Data Mining, San Francisco, CA, USA, August 13–17, 2016*. 2016, 785–94. ACM.

[btad664-B9] Chen T , He T, Benesty M et al Xgboost: extreme gradient boosting. R Package Version 0.4-2. 2015;1:1–4.

[btad664-B10] Chung J , Gulcehre C, Cho K et al Empirical evaluation of gated recurrent neural networks on sequence modeling. arXiv, arXiv:1412.3555, 2014, preprint: not peer reviewed.

[btad664-B11] Cobb M. 60 years ago, Francis Crick changed the logic of biology. PLoS Biol 2017;15:e2003243. 28922352 10.1371/journal.pbio.2003243 PMC5602739

[btad664-B12] Dao F-Y , Lv H, Fullwood MJ et al Accurate identification of DNA replication origin by fusing epigenomics and chromatin interaction information. Research 2022;2022:9780293. 36405252 10.34133/2022/9780293 PMC9667886

[btad664-B13] Dao F-Y , Lv H, Zulfiqar H et al A computational platform to identify origins of replication sites in eukaryotes. Brief Bioinform 2021;22:1940–50. 32065211 10.1093/bib/bbaa017

[btad664-B14] Douglas ME , Ali FA, Costa A et al The mechanism of eukaryotic CMG helicase activation. Nature 2018;555:265–8. 29489749 10.1038/nature25787 PMC6847044

[btad664-B15] Du Q , Bert SA, Armstrong NJ et al Replication timing and epigenome remodelling are associated with the nature of chromosomal rearrangements in cancer. Nat Commun 2019;10:416. 30679435 10.1038/s41467-019-08302-1 PMC6345877

[btad664-B16] Gao F , Luo H, Zhang C-T. DeOri: a database of eukaryotic DNA replication origins. Bioinformatics 2012;28:1551–2. 22467915 10.1093/bioinformatics/bts151

[btad664-B17] Gao F , Zhang C-T. Ori-Finder: a web-based system for finding oriCs in unannotated bacterial genomes. BMC Bioinformatics 2008;9:79. 18237442 10.1186/1471-2105-9-79 PMC2275245

[btad664-B18] He S , Gao B, Sabnis R et al Nucleic transformer: deep learning on nucleic acids with self-attention and convolutions. bioRxiv, 2021–01, 2021, preprint: not peer reviewed.

[btad664-B19] Hochreiter S , Schmidhuber J. Long short-term memory. Neural Comput 1997;9:1735–80. 9377276 10.1162/neco.1997.9.8.1735

[btad664-B20] Hussain S , Mustafa MW, Jumani TA et al A novel feature engineered-CatBoost-based supervised machine learning framework for electricity theft detection. Energy Rep 2021;7:4425–36.

[btad664-B21] Jaganathan K , Rehman MU, Tayara H et al XML-CIMT: explainable machine learning (XML) model for predicting chemical-induced mitochondrial toxicity. Int J Mol Sci 2022;23:15655. 36555297 10.3390/ijms232415655 PMC9779353

[btad664-B22] Ke G , Meng Q, Finley T et al LightGBM: a highly efficient gradient boosting decision tree. In: *Advances in Neural Information Processing Systems, Long Beach Convention & Entertainment Center, Dec 4, 2017 – Dec 9, 2017*, Vol. 30. 2017. MIT Press.

[btad664-B23] Li J , Pu Y, Tang J et al DeepATT: a hybrid category attention neural network for identifying functional effects of DNA sequences. Brief Bioinform 2021;22:bbaa159. 32778871 10.1093/bib/bbaa159

[btad664-B24] Liu B , Weng F, Huang D-S et al iRO-3wPseKNC: identify DNA replication origins by three-window-based PseKNC. Bioinformatics 2018;34:3086–93. 29684124 10.1093/bioinformatics/bty312

[btad664-B25] Lundberg SM , Lee S-I. A unified approach to interpreting model predictions. In: *Advances in Neural Information Processing Systems, Long Beach Convention & Entertainment Center, Dec 4, 2017 – Dec 9, 2017*, Vol. 30. 2017. MIT Press.

[btad664-B26] Luo H , Zhang C-T, Gao F. Ori-Finder 2, an integrated tool to predict replication origins in the archaeal genomes. Front Microbiol 2014;5:482. 25309521 10.3389/fmicb.2014.00482 PMC4164010

[btad664-B27] MacAlpine DM , Almouzni G. Chromatin and DNA replication. Cold Spring Harb Perspect Biol 2013;5:a010207. 23751185 10.1101/cshperspect.a010207 PMC3721285

[btad664-B28] Moiseeva TN , Bakkenist CJ. Regulation of the initiation of DNA replication in human cells. DNA Repair (Amst) 2018;72:99–106. 30266203 10.1016/j.dnarep.2018.09.003 PMC6261693

[btad664-B29] Norris V. Does the semiconservative nature of DNA replication facilitate coherent phenotypic diversity? J Bacteriol 2019;201:e00119. 30936370 10.1128/JB.00119-19 PMC6531617

[btad664-B30] Prokhorenkova L , Gusev G, Vorobev A et al CatBoost: unbiased boosting with categorical features. In: *Advances in Neural Information Processing Systems, Montreal Convention Centre, Dec 3, 2018 – Dec 8, 2018*, Vol. 31. 2018. MIT Press.

[btad664-B31] Singh VK , Kumar V, Krishnamachari A. Prediction of replication sites in *Saccharomyces cerevisiae* genome using DNA segment properties: multi-view ensemble learning (MEL) approach. Biosystems 2018;163:59–69. 29233729 10.1016/j.biosystems.2017.12.005

[btad664-B32] Vaswani A , Shazeer N, Parmar N et al Attention is all you need. In: *Advances in Neural Information Processing Systems, Long Beach Convention & Entertainment Center, Dec 4, 2017 – Dec 9, 2017*, Vol. 30. 2017. MIT Press.

[btad664-B33] Wei L , He W, Malik A et al Computational prediction and interpretation of cell-specific replication origin sites from multiple eukaryotes by exploiting stacking framework. Brief Bioinform 2021;22:bbaa275. 33152766 10.1093/bib/bbaa275

[btad664-B34] Yao Z , Zhang W, Song P et al DeepFormer: a hybrid network based on convolutional neural network and flow-attention mechanism for identifying the function of DNA sequences. Brief Bioinform 2023;24:bbad095. 36917472 10.1093/bib/bbad095

[btad664-B35] Zhang C-J , Tang H, Li W-C et al iOri-Human: identify human origin of replication by incorporating dinucleotide physicochemical properties into pseudo nucleotide composition. Oncotarget 2016;7:69783–93. 27626500 10.18632/oncotarget.11975 PMC5342515

