# Supplementary material for: ORI-Explorer: a unified cell-specific tool for origin of replication sites prediction by feature fusion
Source: Bioinformatics. 2023 Oct 31;39(11):btad664. doi: 10.1093/bioinformatics/btad664 (PMC10639035; doi:10.1093/bioinformatics/btad664)
Supplement: btad664_Supplementary_Data [file btad664_supplementary_data.pdf]

Table S1: Summary of the used training and independent datasets

Species	Cell	Training		Independent	
		ORIs (pos)	Non-ORIs (neg)	ORIs (pos)	Non-ORIs (neg)
H. sapiens	K562	1332	1331	1000	1000
	MCF7	1763	1763	1000	1000
M. musculus	ES	1380	1380	1000	1000
	MEF	1202	1202	1000	1000
	P19	1725	1725	1000	1000
D. melanogaster	Kc	3000	3000	3000	3000
	Bg3	2763	2764	2000	2000
	S2	3550	3551	3000	3000
A. thaliana	—	1015	1015	500	500

Table S2: Hyperparameter ranges used in DL and CatBoost Classifier

	Parameters	Ranges
**DL module**	Convolution filters	[8,16,32,64,96,128]
	Bi-GRU units	[8,16,32,64,96,128]
	Dense nodes	[8,16,32,64,96,128]
**CatBoost Classifier**	learning rate	Float (0.01 - 0.5)
	Max depth	[2,3,4,5,6,7,8,9,10]
	Boosting type	['ordered', 'plain']
	l2_leaf_reg	[2,3,4,5,6,7,8,9,10]
	random strength	[0,1,2,3,4,5,6,7,8,9,10]

Table S3: Selected parameters in DL module (lr: learning_rate, C1-C6: conv1 – conv6, D1 & D2: Dense1 & Dense2)

**Specie**	**Cell**	**lr**	**conv1**	**conv2**	**conv3**	**conv4**	**conv5**	**conv6**	**GRU1**	**GRU2**	**D1**	**D2**
H.sapiens	K562	0.0003346585347938224	64	96	32	16	32	96	96	16	32	16
	MCF7	0.00033465854	64	96	32	16	32	96	96	16	32	16
M.musculus	ES	0.0003346585347938224	64	96	32	16	32	96	96	16	32	16
	MEF	0.0004528658202015476	96	128	32	8	96	32	64	16	96	96
	P19	0.0001219643973048492	128	8	16	96	8	96	32	96	128	64
D. melanogaster	KC	0.00011730817269837467	96	32	8	16	128	32	32	96	64	64
	BG3	0.00012004313925889691	96	32	128	32	32	16	16	128	128	128
	S2	0.00010477816811921633	8	16	64	32	96	8	128	64	16	16
A.thaliana	-	0.00019007194589958247	128	64	16	96	128	128	96	64	128	128

Table 4: Selected parameters in CatBoost Classifier (lr: learning_rate, md: max_depth, b_type: boosting type, lr_leaf: l2_leaf_reg, r_str: random_strength)

Specie	Cell	lr	md	b_type	lr_leaf	r_str
H.sapiens	K562	0.022561333400184586	6	Plain	2	8
	MCF7	0.03961800783872605	9	Plain	4	4
M.musculus	ES	0.076275702658	9	Plain	6	1
	MEF	0.0220834969	2	Plain	10	7
	P19	0.04458134418270355	10	Plain	5	3
D. melanogaster	KC	0.0523224109689 1192	6	Plain	6	3
	BG3	0.03175707636199941	9	Plain	10	8
	S2	0.04504523555372621	6	Plain	9	9
A.thaliana	AT	0.20806277456059852	9	Plain	8	6

Table S5: Performance assessment on individual feature encodings

Specie	Cell	Encoding	MCC	Acc	Sn	Sp	AUC	F1	Precision
H. sapiens	K562	CKSNAP	0.738	0.869	0.848	0.890	0.941	0.866	0.885
		PCPseDNC	0.698	0.849	0.843	0.855	0.927	0.848	0.853
		DCC	0.624	0.812	0.808	0.816	0.899	0.811	0.815
		DL	0.726	0.863	0.846	0.88	0.934	0.861	0.876
	MCF7	CKSNAP	0.609	0.805	0.782	0.827	0.90	0.80	0.819
		PCPseDNC	0.583	0.792	0.774	0.809	0.89	0.788	0.802
		DCC	0.546	0.773	0.751	0.795	0.853	0.768	0.786
		DL	0.610	0.804	0.775	0.834	0.898	0.799	0.824
M. musculus	ES	CKSNAP	0.689	0.845	0.831	0.858	0.913	0.842	0.854
		PCPseDNC	0.662	0.831	0.82	0.842	0.906	0.829	0.838
		DCC	0.546	0.773	0.763	0.783	0.856	0.771	0.779
		DL	0.746	0.873	0.875	0.871	0.949	0.873	0.872
	MEF	CKSNAP	0.545	0.772	0.746	0.798	0.865	0.765	0.786
		PCPseDNC	0.558	0.779	0.763	0.795	0.866	0.775	0.788
		DCC	0.489	0.744	0.701	0.787	0.821	0.732	0.767
		DL	0.667	0.834	0.808	0.859	0.892	0.829	0.851
	P19	CKSNAP	0.591	0.795	0.802	0.789	0.879	0.796	0.791
		PCPseDNC	0.589	0.794	0.797	0.792	0.874	0.795	0.793
		DCC	0.499	0.749	0.745	0.754	0.829	0.748	0.751
		DL	0.720	0.860	0.869	0.851	0.935	0.861	0.854
D. melanogaster	KC	CKSNAP	0.692	0.846	0.861	0.830	0.919	0.848	0.836
		PCPseDNC	0.649	0.824	0.839	0.809	0.899	0.827	0.815
		DCC	0.549	0.774	0.799	0.749	0.852	0.779	0.761
		DL	0.688	0.843	0.868	0.819	0.921	0.848	0.828
	BG3	CKSNAP	0.497	0.748	0.733	0.764	0.830	0.744	0.756
		PCPseDNC	0.459	0.729	0.719	0.739	0.799	0.727	0.734
		DCC	0.342	0.671	0.664	0.679	0.736	0.669	0.674
		DL	0.680	0.840	0.854	0.827	0.920	0.842	0.831
	S2	CKSNAP	0.420	0.710	0.690	0.730	0.777	0.704	0.718

		PCPseDNC	0.342	0.671	0.654	0.689	0.735	0.665	0.677
		DCC	0.249	0.625	0.615	0.634	0.682	0.621	0.627
		DL	0.614	0.806	0.839	0.773	0.877	0.813	0.787
A. thaliana	-	CKSNAP	0.751	0.875	0.902	0.848	0.948	0.878	0.855
		PCPseDNC	0.773	0.886	0.908	0.864	0.950	0.888	0.869
		DCC	0.666	0.833	0.848	0.818	0.914	0.835	0.823
		DL	0.856	0.928	0.936	0.920	0.977	0.928	0.921

Table S6: Performance assessment without attention and Bi-GRU modules

**Specie**	**Cell**	**Method**	**MCC**	**Acc**	**Sn**	**Sp**	**AUC**	**F1**	**Precision**
H. sapiens	K562	Complete network	0.769	0.884	0.851	0.917	0.950	0.880	0.911
		Without attention	0.748	0.874	0.859	0.889	0.949	0.8720	0.885
		Without Bi-GRU	0.676	0.838	0.828	0.848	0.918	0.836	0.845
	MCF7	Complete network	0.675	0.836	0.791	0.881	0.919	0.828	0.869
		Without attention	0.612	0.806	0.787	0.825	0.898	0.802	0.818
		Without Bi-GRU	0.569	0.785	0.769	0.80	0.879	0.781	0.794

Table S7: Features Range showing which features belongs to which encoding

**Specie**	**Cell**	**DL Features**	**CKSNAP Features**	**PCPseDNC Features**	**DCC Features**
H.sapiens	K562	16	96	18	60
	MCF7	16	96	18	60
M.musculus	ES	16	96	18	60
	MEF	96	96	18	60
	P19	64	96	18	60
D. melanogaster	KC	64	96	18	60
	BG3	128	96	18	60
	S2	16	96	18	60
A.thaliana	-	128	96	18	60

Since we optimized the network using Optuna, the number of nodes in the last layer for each cell is different, causing variable number of features for each cell. The number of DL features are mentioned in

DL Features column in table above. The other number of features are fixed since they are extracted using ML feature extraction techniques. So, the range of features for each cell can be defined as:

K562 → 0 —DL→ 15 —CKSNAP→ 111 —PCPseDNC→ 129 —DCC→ 189

MCF7 → 0 —DL→ 15 —CKSNAP→ 111 —PCPseDNC→ 129 —DCC→ 189

ES → 0 —DL→ 15 —CKSNAP→ 111 —PCPseDNC→ 129 —DCC→ 189

MEF → 0 —DL→ 95 —CKSNAP→ 191 —PCPseDNC→ 209 —DCC→ 269

P19 → 0 —DL→ 63 —CKSNAP→ 159 —PCPseDNC→ 177 —DCC→ 237

KC → 0 —DL→ 63 —CKSNAP→ 159 —PCPseDNC→ 177 —DCC→ 237

BG3 → 0 —DL→ 127 —CKSNAP→ 223 —PCPseDNC→ 241 —DCC→ 301

S2 → 0 —DL→ 15 —CKSNAP→ 111 —PCPseDNC→ 129 —DCC→ 189

Thaliana → 0 —DL→ 127 —CKSNAP→ 223 —PCPseDNC→ 241 —DCC→ 301
